# CDC5L promotes early chondrocyte differentiation and proliferation by modulating pre-mRNA splicing of *SOX9*, *COL2A1*, and *WEE1*

**DOI:** 10.1016/j.jbc.2021.100994

**Published:** 2021-07-21

**Authors:** Go Jokoji, Shingo Maeda, Kazuki Oishi, Toshiro Ijuin, Masahiro Nakajima, Hiroki Tawaratsumida, Ichiro Kawamura, Hiroyuki Tominaga, Eiji Taketomi, Shiro Ikegawa, Noboru Taniguchi

**Affiliations:** 1 Department of Bone and Joint Medicine, Graduate School of Medical and Dental Sciences, Kagoshima University, Kagoshima, Japan; 2 Department of Orthopaedic Surgery, Graduate School of Medical and Dental Sciences, Kagoshima University, Kagoshima, Japan; 3 Laboratory for Bone and Joint Diseases, RIKEN Center for Integrative Medical Sciences, Tokyo, Japan; 4 Department of Orthopaedic Surgery, Japanese Red Cross Kagoshima Hospital, Kagoshima Japan

**Keywords:** ossification of the posterior longitudinal ligament (OPLL), chondrocyte, cell division cycle 5-like (CDC5L), Wee1, cell cycle, mRNA splicing, Agc1, aggrecan, CDC5L, cell division cycle 5-like, COL II, type II collagen, DMEM, Dulbecco's modified Eagle's medium, eQTL, expression quantitative trait loci, GFP, green fluorescent protein, FBS, fetal bovine serum, GWAS, genome-wide association study, IF, immunofluorescence, IHC, immunohistochemistry, ITS, insulin/transferrin/selenium, MSC, mesenchymal stem cell, OPLL, ossification of the posterior longitudinal ligament, PCNA, proliferating cell nuclear antigen, PTHrP, parathyroid-hormone-related protein, SDS, sodium dodecyl sulfate, SNP, single nucleotide polymorphism, TGF, transforming growth factor, TUNEL, terminal deoxynucleotidyl transferase dUTP nick-end labeling, WST, water-soluble tetrazolium

## Abstract

Ossification of the posterior longitudinal ligament (OPLL) of the spine is a common pathological condition that causes intractable myelopathy and radiculopathy, mainly the result of an endochondral ossification-like process. Our previous genome-wide association study identified six susceptibility loci for OPLL, including the cell division cycle 5-like (CDC5L) gene region. Here, we found CDC5L to be expressed in type II collagen-producing chondrocyte-like fibroblasts in human OPLL specimens, as well as in differentiating ATDC5 chondrocytes. *Cdc5l* siRNA transfection in murine chondrocytes decreased the expression of the early chondrogenic genes *Sox9* and *Col2a1*, diminished the cartilage matrix production, and enhanced the expression of parathyroid-hormone-related protein (a resting chondrocyte marker). We also showed that *Cdc5l* shRNA suppressed the growth of cultured murine embryonal metatarsal cartilage rudiments and that *Cdc5l* knockdown suppressed the growth of ATDC5 cells. Fluorescence-activated cell sorting analysis revealed that the G2/M cell cycle transition was blocked; our data showed that *Cdc5l* siRNA transfection enhanced expression of Wee1, an inhibitor of the G2/M transition. *Cdc5l* siRNA also decreased the pre-mRNA splicing efficiency of *Sox9* and *Col2a1* genes in both ATDC5 cells and primary chondrocytes; conversely, loss of Cdc5l resulted in enhanced splicing of *Wee1* pre-mRNA. Finally, an RNA-binding protein immunoprecipitation assay revealed that Cdc5l bound directly to these target gene transcripts. Overall, we conclude that Cdc5l promotes both early chondrogenesis and cartilage growth and may play a role in the etiology of OPLL, at least in part by fine-tuning the pre-mRNA splicing of chondrogenic genes and *Wee1*, thus initiating the endochondral ossification process.

Ossification of the posterior longitudinal ligament (OPLL) of the spine is a common pathological condition characterized by ectopic bone formation within the spinal posterior longitudinal ligament (PLL) in the spinal canal; this compresses the spinal cord and nerve root to cause severe myelopathy and root dysfunction (1). OPLL affects 1.9–4.3% of the Japanese population, 0.4–3.0% of other Asian populations, and 0.1–1.7% of the Caucasian population (2). To date, surgical treatment that decompresses the spinal cord is the only option to relieve severe neuropathy; however, surgical outcomes are often unsatisfactory because of irreversible spinal nerve damage resulting from long-term compression. Therefore, there is an urgent need to develop an alternative nonsurgical approach to prevent or control OPLL growth. Histological studies have indicated that OPLL is formed mainly through a process similar to endochondral ossification (3, 4, 5, 6); we had observed the cartilage-specific type II and XI collagen expression in the cartilage-like area adjacent to the ossification front (7). In addition, we recently confirmed this concept through immunohistochemically evaluating the expression of a series of proteins specific to endochondral ossification around the ossification area of OPLL (8). Endochondral bone formation is initiated by condensation of mesenchymal progenitor cells expressing the chondrogenic master transcription factor Sox9, which drives the expression of cartilage-specific matrix proteins such as type II collagen (COL II, Col2a1) and aggrecan (Agc1) in chondrocytes (9). Resting (nonproliferating) chondrocytes, specifically expressing parathyroid-hormone-related protein (PTHrP), are skeletal stem cells that undergo chondrocyte differentiation and proliferation (10). PTHrP maintains chondrocytes in a proliferative state, which is indispensable for endochondral bone growth (11, 12). As the distance from PTHrP-expressing resting chondrocytes increases, the columnar chondrocytes stop proliferating and differentiate into type X collagen (COL X, Col10a1)-expressing hypertrophic chondrocytes, mineralizing their surrounding matrix. Then, hypertrophic chondrocytes attract blood vessels and stimulate chondroclasts to migrate. The chondroclasts degrade mineralized cartilage matrix to provide space for osteoblastic bone formation (13). However, to date, the molecular mechanisms that initiate chondrogenesis in spinal ligaments and cause OPLL remain largely elusive.

Genetic background is thought to be related to OPLL onset or progression (14, 15, 16, 17). Groups of the investigation committee on the ossification of spinal ligaments, including our laboratories, have previously performed a genome-wide association study (GWAS) in the Japanese population to identify six susceptibility loci for OPLL: 20p12.3, 8q23.1, 12p11.22, 12p12.2, 8q23.3, and 6p21.1 (16). However, it is unclear which of the genes within the loci result in substantial protein levels that contribute to the pathogenesis of OPLL, especially during endochondral ossification.

In this study, we focused on one of the 6p21.1 locus resident genes, CDC5L (cell division cycle 5-like), because our FANTOM5 database analysis has previously shown it to be differentially expressed between human osteoblasts and fibroblasts (16). CDC5L is a pre-mRNA splicing factor and a core component of the human Prp19/CDC5L complex that plays an essential role in G2 phase progression and entry into mitosis in the cell cycle (18, 19, 20, 21, 22). CDC5L promotes the expression of a set of genes involved in mitosis (DYNC1H1, DYNLRB2, and DCTN4), and the DNA damage response (RAD1 and BARD1), as the pre-mRNA splicing efficiency of these genes was impaired by CDC5L knockdown (23). As a cell cycle driver, CDC5L is overexpressed in many tumors, including osteosarcoma (24, 25, 26), and plays oncogenic roles (27, 28, 29). However, the expression of CDC5L in OPLL or normal developing cartilage has not been investigated so far. Additionally, there are no reports regarding the impact of CDC5L on cell differentiation. Here, we examined CDC5L expression in OPLL specimens and in mouse embryo bone cartilage samples. We studied the roles of CDC5L in early chondrocyte differentiation and proliferation by modulating pre-mRNA splicing of Sox9, Col2a1, and Wee1.

## Results

### CDC5L is increased in fibroblasts carrying the OPLL risk allele that is expressed in the fibrocartilage area of OPLL

**Figure 1:**
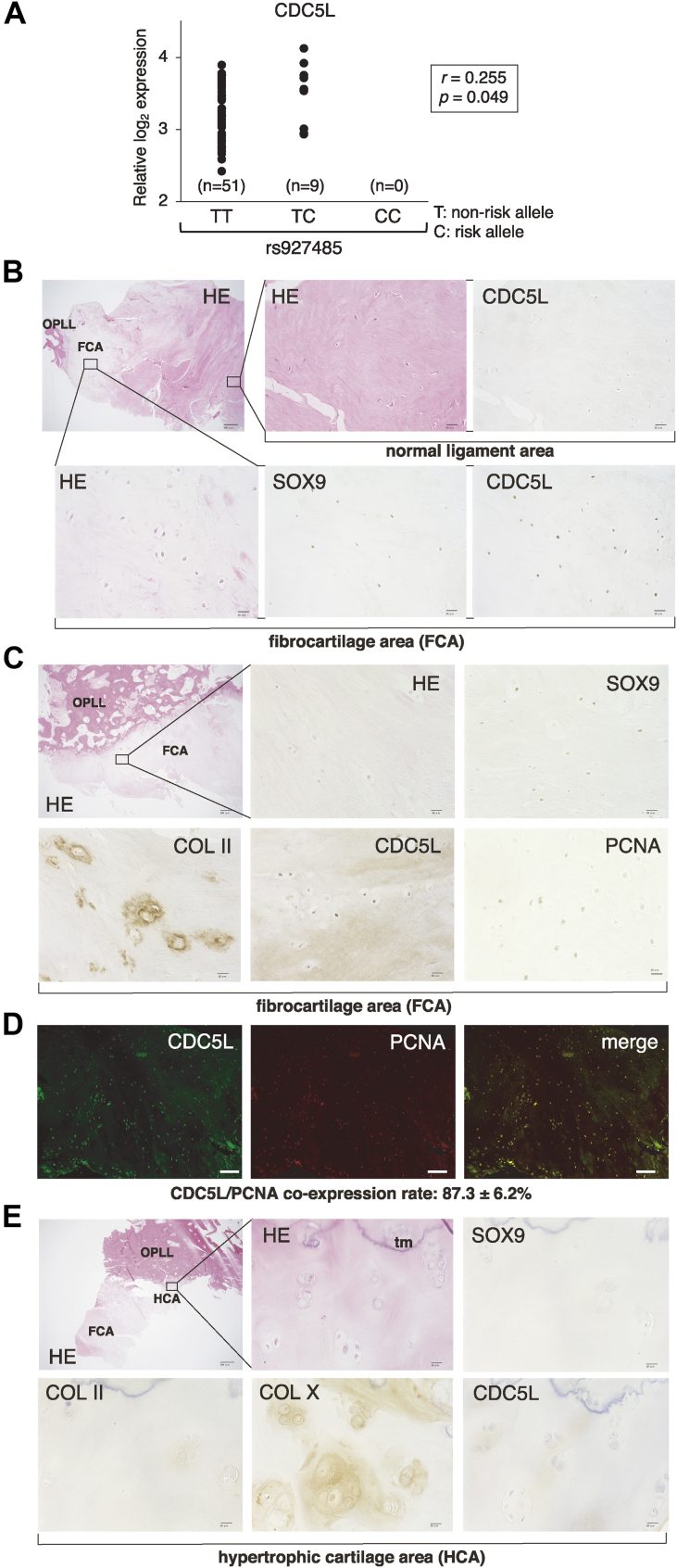
**Cell division cycle 5-like protein (CDC5L) is expressed in the fibrocartilage area of the ossification of the posterior longitudinal ligament (OPLL).***A*, the effect of OPLL susceptibility single nucleotide polymorphism rs927485 on CDC5L expression in human fibroblasts. An expression quantitative trait loci (eQTL) analysis was performed to show that the heterozygous subjects carrying the risk allele C had higher CDC5L expression than homozygous specimens with the non-risk-allele T. *B* and *C*, the human OPLL specimens including the fibrocartilage area were subjected to hematoxylin and eosin (HE) staining and immunohistochemistry for CDC5L, SOX9, COL II, and proliferating cell nuclear antigen (PCNA). Samples used in *B* and *C* were obtained from distinct individuals. Scale bars, 500 μm (*left upper panel*) and 20 μm. *D*, the same OPLL specimen as in C was subjected to double immunofluorescence for CDC5L and PCNA. Scale bars, 50 μm. Numbers of expression-positive cells in four independent fields were counted using the BZ-X710/BZ-X700 microscope system, from which the coexpression rate was calculated. *E*, a human OPLL specimen (from another patient) including the hypertrophic cartilage area was subjected to HE staining and immunohistochemistry for SOX9, COL II, COL X, and CDC5L. Scale bars, 500 μm (*left upper panel*) and 20 μm.

First, to test whether the OPLL-associated single nucleotide polymorphism (SNP) rs927485, residing adjacent to the CDC5L gene in locus 6p21.1 (16), actually affects CDC5L expression in human cells, we conducted an expression quantitative trait loci (eQTL) analysis using normal human fibroblast samples. Although the homozygote for the OPLL risk allele C (CC) was not included in our specimens, heterozygous (TC) cells exhibited higher CDC5L expression levels than those of nonrisk allele homozygotes (TT), showing an autosomal dominant effect (Fig. 1*A*). We next assessed with immunohistochemistry (IHC) whether CDC5L is expressed at the protein level in human OPLL tissue. CDC5L protein was not detected in normal unaffected PLL cells (Fig. 1*B*). We have characterized the degenerated fibrocartilage area adjacent to the ossification front of OPLL specimens in which the resident cells express SOX9 and COL II but not COL X, indicating that these cells are in the early chondrocyte differentiation stage (8). In this area, we found substantial CDC5L expression along with SOX9 and COL II staining (Fig. 1, *B* and *C*). As CDC5L is a cell cycle promoter, we examined whether CDC5L-expressing cells were proliferative with IHC of the proliferating cell nuclear antigen (PCNA). We found that these fibrocartilage area cells indeed coexpressed PCNA (Fig. 1*C*). We further performed double immunofluorescence (IF) for CDC5L and PCNA and found a high coexpression rate of 87.3% in the fibrocartilage area cells (Fig. 1*D*). The hypertrophic cartilage area is characterized by the presence of COL X-expressing, enlarged, round, hypertrophied chondrocytes, which are negative for SOX9 and COL II expression. Their location, which is contiguous with the matrix calcification tide mark, indicates that the resident cells are in the late endochondral ossification phase (8) (Fig. 1*E*). As expected, because hypertrophic chondrocytes do not proliferate, CDC5L was not detected in the hypertrophic cartilage area (Fig. 1*E*).

### Cdc5l is expressed in proliferating chondrocytes of mouse embryo cartilage

**Figure 2:**
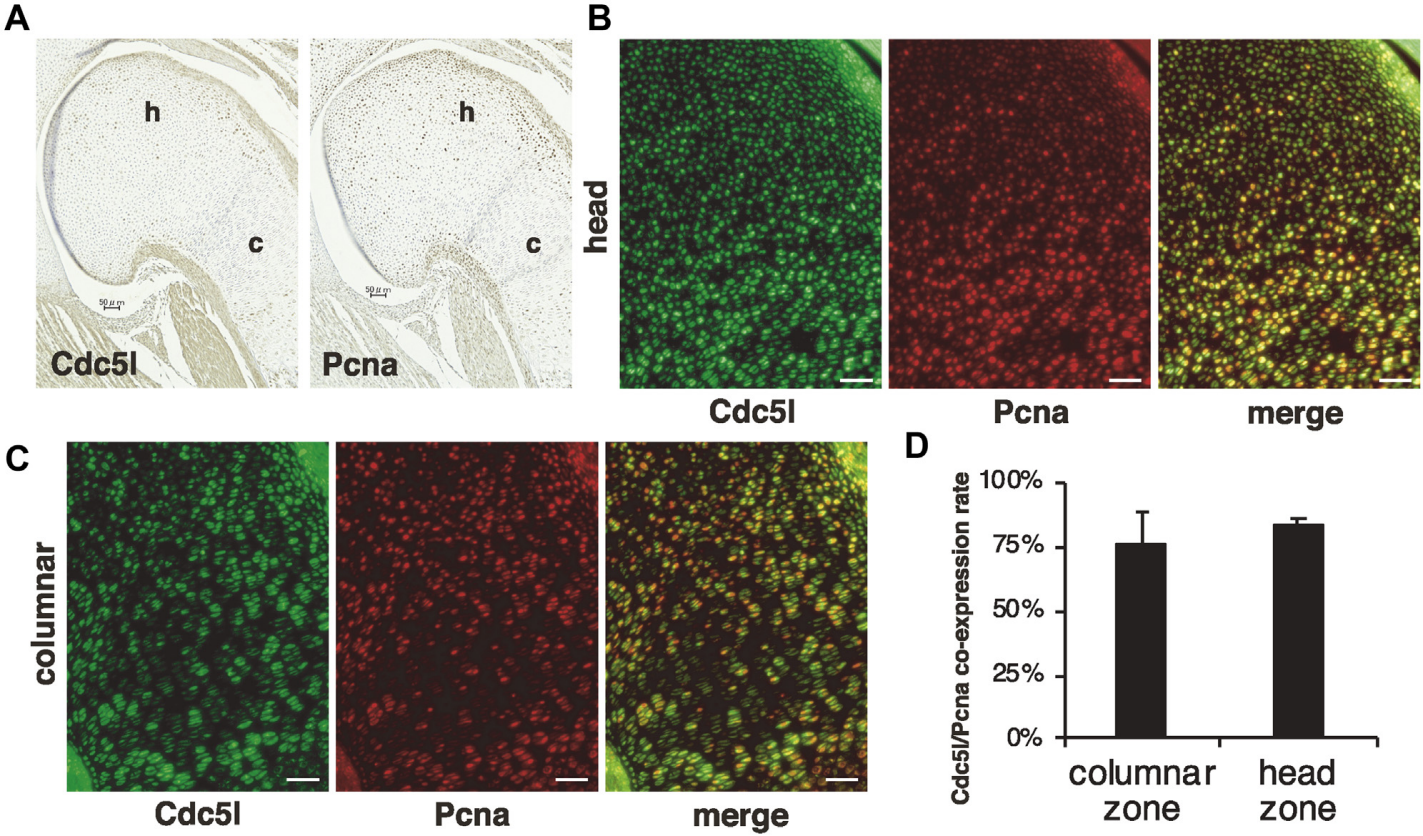
**Cdc5l is expressed in Pcna-positive proliferating chondrocytes in mouse embryo cartilage.***A*, humeral bone rudiment of mouse E17.5 embryo was subjected to immunohistochemistry for Cdc5l and Pcna. Scale bars, 50 μm. *B* and *C*, double immunofluorescence for Cdc5l and Pcna was performed. Fields of humeral head (h) and columnar (c) proliferative chondrocytes are presented. Scale bars, 50 μm. *D*, Numbers of expression-positive cells in four independent fields each of humeral columnar or head zones were counted, and the Cdc5l/Pcna coexpression rate was calculated.

We next examined mouse Cdc5l protein expression under physiological endochondral ossification conditions using mouse humerus specimens from embryonic day 17.5 (E17.5). Cdc5l was detected not only in the columnar proliferating chondrocytes, as expected, but also in the round chondrocytes in the humeral head (Fig. 2*A*). These Cdc5l-positive cells were in the proliferating state, as determined by IHC and IF of Pcna (Fig. 2, *A*–*C*), with a coexpression rate exceeding 75% (Fig. 2*D*). These IHC/IF results in OPLL and mouse embryo bone specimens suggest the roles of CDC5L in the early phase of endochondral ossification.

### Cdc5l knockdown blocks G2/M transition to suppress chondrocyte growth and enhances Wee1 expression

**Figure 3:**
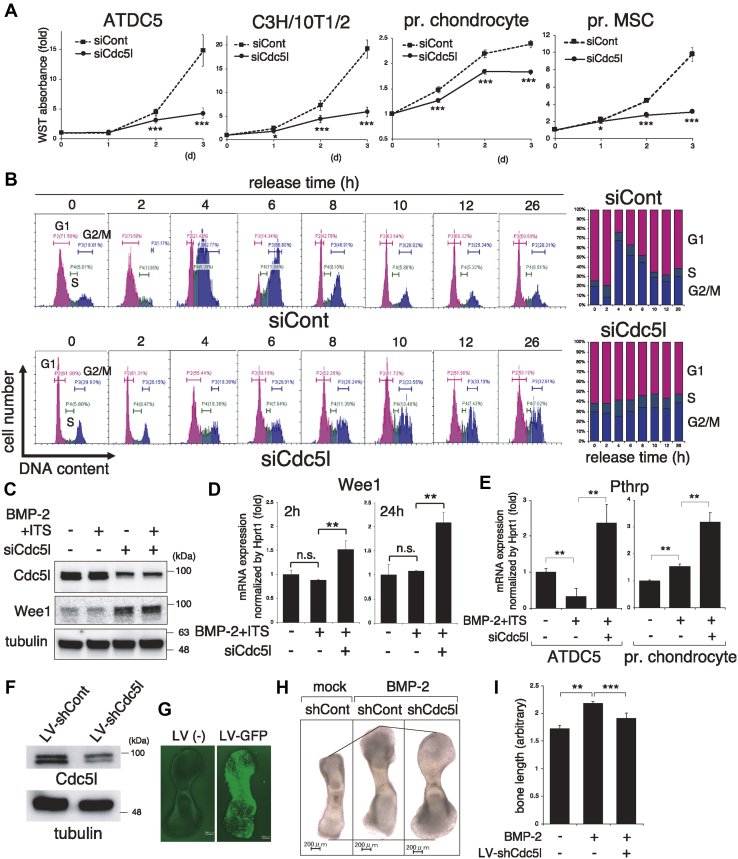
**Cdc5l knockdown abrogates chondrocyte growth and enhances Wee1 expression.***A*, ATDC5, C3H/10T1/2 cells, primary (pr.) chondrocytes, and primary mesenchymal stem cells (MSCs) were transfected with control or Cdc5l siRNA overnight. Live cell numbers were monitored by water-soluble tetrazolium (WST) assay daily for 3 days (n = 8). *B*, a time course study of the cell cycle. ATDC5 cells transfected with siCont or siCdc5l were released from G1/S synchronization, induced by thymidine double block, and subjected to fluorescence-activated cell sorting analysis at the indicated time points (a representative data set from the triplicate experiments, *left panels*). The average percentages of cell numbers in each cell cycle phase are presented (n = 3, *right panels*). *C* and *D*, ATDC5 cells were transfected with siCont or siCdc5l overnight, followed by application of BMP-2 + ITS. Immunoblotting with indicated antibodies was performed at 2 h (*C*). Tubulin served as a loading control. RT-qPCR for Wee1 was analyzed at 2 h and 24 h (n = 3) (*D*). *E*, ATDC5 cells or primary (pr.) chondrocytes were transfected with siCont or siCdc5l and stimulated with BMP-2 + ITS. RT-qPCR for parathyroid-hormone-related protein was performed on day 7 (ATDC5, n = 3) or day 6 (pr. chondrocytes, n = 3). *F*, ATDC5 cells were infected with LV-shCont or LV-shCdc5l overnight and cultured in complete medium for 2 days. The knockdown efficiency was evaluated by immunoblot against Cdc5l. Tubulin served as a loading control. *G–I*, E15.5 mouse metatarsal bones were infected with or without lentiviral particles carrying GFP or indicated shRNA and cultured with or without BMP-2 (300 ng/ml) for 5 days. LV-infection efficiency in the cartilage organ culture was evaluated by the green signal of GFP-control LV (*G*). Total bone length was measured by analyzing the captured images (*H* and *I*) (n = 5). Scale bars, 200 μm. ∗*p* < 0.05; ∗∗*p* < 0.01; ∗∗∗*p* < 0.001; n.s., not significant.

To test the role of Cdc5l in chondrocyte growth as a G2/M transition promoter, we performed water-soluble tetrazolium (WST)-1 cell proliferation assay with Cdc5l siRNA transfection in the mouse chondrogenic ATDC5 cell line, mouse embryonic fibroblast C3H/10T1/2 cells, mouse primary chondrocytes, and mouse primary mesenchymal stem cells (MSCs). Cdc5l knockdown suppressed the growth of all the tested cells (Fig. 3*A*). To examine whether this was due to inhibited G2/M transition, we synchronized ATDC5 cells at the G1/S boundary using double thymidine block methods, released cells into the cell cycle by removing thymidine, and performed flow cytometry assay. The control siRNA-treated cells progressed to the cell cycle, accumulated in the G2/M phase 4 h after release, and subsequently moved into G1 phase after 8 h (Fig. 3*B*). However, siCdc5l-treated cells reached the peak of the G2/M phase 8 h after release and remained in the G2/M phase thereafter (Fig. 3*B*), suggesting that siCdc5l-mediated cell growth retardation was due to, at least in part, hindered G2/M transition. We next investigated whether the expression level of Wee1, the well-known G2/M transition inhibitor (30), was affected by siCdc5l. We transfected Cdc5l siRNA into ATDC5 cells overnight and stimulated cells with bone morphogenetic protein (BMP)-2 plus insulin/transferrin/selenium (ITS) supplement (for chondrogenesis induction) for 2 h, then confirmed the knockdown efficiency by immunoblotting (Fig. 3*C*). Anti-Cdc5l antibody detected double bands just below the 100 kDa marker, while siRNA eliminated the lower band intensity, indicating the lower band to be the Cdc5l signal. We found that the Wee1 band was significantly strengthened upon siCdc5l induction (Fig. 3*C*). The 2 h stimulation with BMP-2+ITS did not alter either of the protein bands. We obtained similar results of Wee1 upregulation at the mRNA level from reverse transcription quantitative polymerase chain reaction (RT-qPCR) analysis both 2 h and 24 h after BMP-2+ITS stimulation in ATDC5 cells (Fig. 3*D*). Next, we examined whether siCdc5l leads chondrocytes to the resting state and found accumulated PTHrP expression by Cdc5l knockdown, both in ATDC5 cells and primary chondrocytes (Fig. 3*E*). To further examine the role of Cdc5l in cell growth under relatively more physiological chondrogenic conditions, we employed an *ex vivo* organ culture system of mouse embryonic (E15.5) metatarsal bone cartilage rudiment. This is a widely used method that permits the study of a complex chondrogenic process in a three-dimensional structure in the context of native cell–cell and cell–extracellular matrix interactions and cellular signaling (31). For Cdc5l knockdown in organ cultures, we employed a lentivirus (LV) system to induce short hairpin (sh) RNA for gene silencing. We assessed the knockdown efficiency of LV-shCdc5l in ATDC5 cells to determine the substantial elimination of the lower band by immunoblot (Fig. 3*F*). We then assessed the LV infection efficiency in the cartilage culture using green fluorescent protein (GFP)-expressing LV to confirm sufficient green signal induction throughout the cartilage (Fig. 3*G*). BMP-2 enlarged the cultured cartilage after 5 days, while LV-shCdc5l infection negated this effect (Fig. 3*H*). We measured the cartilage length and found that the LV-shCdc5l-induced growth inhibition was statistically significant (Fig. 3*I*).

### CDC5L overexpression or Wee1 knockdown rescues siCdc5l-mediated cell growth retardation in ATDC5 cells

**Figure 4:**
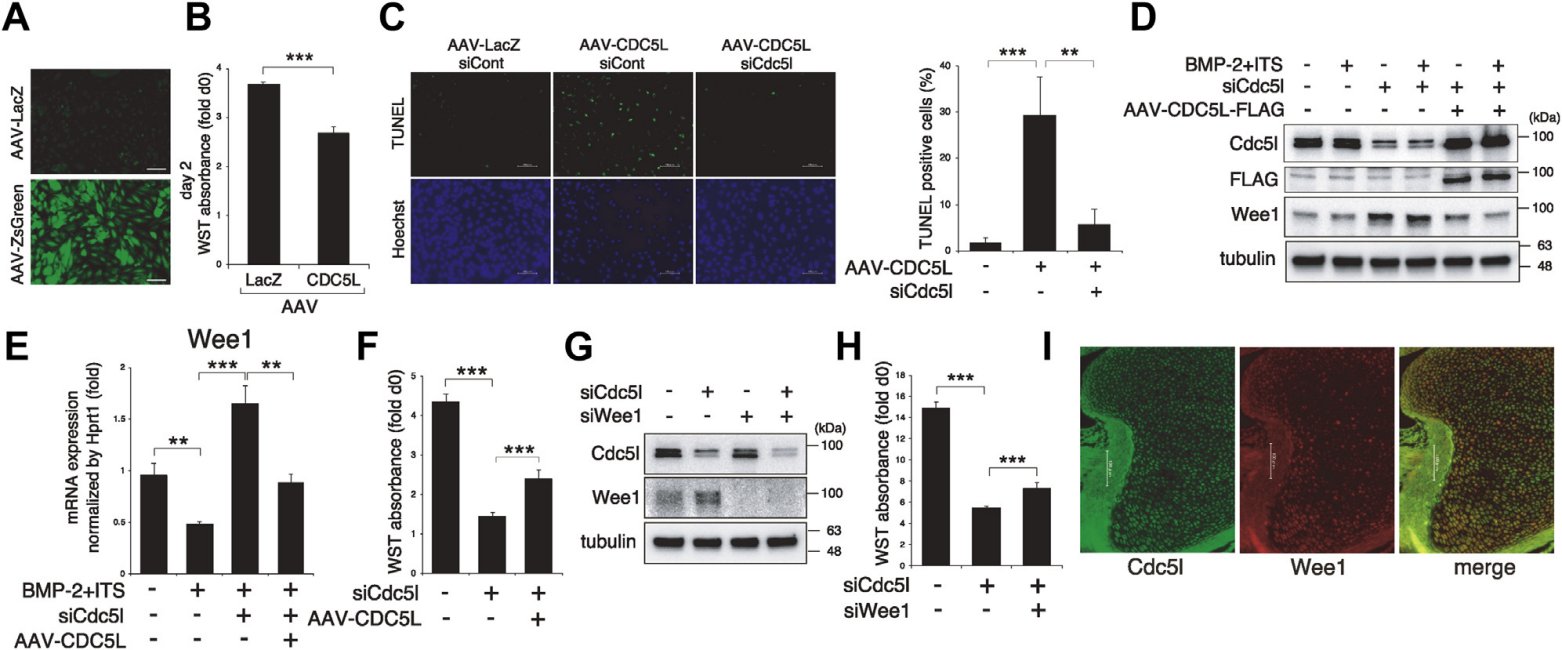
**Overexpression of CDC5L or Wee1 knockdown partially rescues the siCdc5l-mediated growth retardation in ATDC5.***A*, ATDC5 cells were infected with adeno-associated virus (AAV)-LacZ or AAV-ZsGreen for 2 days. The infection efficiency was evaluated by the green signal of ZsGreen protein. Scale bars, 100 μm. *B*, ATDC5 cells were infected with AAV-LacZ or AAV-CDC5L, and the cell number was monitored by water-soluble tetrazolium (WST) assay on day 2 (n = 8). *C*, ATDC5 cells transfected with indicated siRNA were infected with AAV-LacZ or AAV-CDC5L for 2 days and subjected to fluorescent TUNEL assay. Scale bars, 100 μm. The percentage of TUNEL-positive cells normalized by the number of Hoechst-stained cells are shown in the *right panel*. *D*, ATDC5 cells transfected with siCont or siCdc5l were infected with AAV-LacZ or AAV-CDC5L for 2 days. Then cells were stimulated with or without BMP-2 + ITS for 2 h to be subjected to immunoblotting with indicated antibodies. Tubulin served as a loading control. *E*, ATDC5 cells transfected with siCont or siCdc5l were infected with AAV-LacZ or AAV-CDC5L for 2 days, followed by BMP-2 + ITS application for 4 days. RT-qPCR for Wee1 was analyzed (n = 3). *F*, ATDC5 cells transfected with siCont or siCdc5l were infected with AAV-LacZ or AAV-CDC5L, and cell number was evaluated by WST assay on day 4 (n = 8). *G*, ATDC5 cells transfected with siCont or siCdc5l were cultured for 2 days to be subjected to immunoblotting with indicated antibodies. Tubulin served as a loading control. *H*, ATDC5 cells transfected with combinations of siCont, siCdc5l, and siWee1 were subjected to WST assay on day 3 (n = 8). *I*, double immunofluorescence for Cdc5l and Wee1 was performed on E17.5 mouse humerus specimen. Scale bars, 100 μm. ∗∗*p* < 0.01; ∗∗∗*p* < 0.001.

To investigate the effects of increased Cdc5l function, we employed an adeno-associated virus (AAV) system to overexpress the human CDC5L gene. We assessed the infection efficiency of the AAV systems of all the serotypes by infecting ZsGreen protein-expressing corresponding AAVs and found that serotype 6 is the best for infecting ATDC5 cells (Fig. 4*A*). Unexpectedly, AAV-CDC5L inhibited ATDC5 cell growth (Fig. 4*B*). We postulated whether apoptosis was involved in this process, as dysregulated cell cycles often result in cell death. With the fluorescent terminal deoxynucleotidyl transferase dUTP nick-end labeling (TUNEL) assay, we found that the forced CDC5L expression substantially induced apoptosis in ATDC5 cells (Fig. 4*C*). This apoptosis was eliminated by coinduction of Cdc5l siRNA, suggesting that excess CDC5L expression above physiological levels evokes apoptosis (Fig. 4*C*). Accordingly, we designed a rescue experiment to further examine the gain-of-function effect. We infected AAV-CDC5L in siCdc5l transfected cells and confirmed with immunoblotting that induced CDC5L protein levels were comparable with endogenous Cdc5l levels (Fig. 4*D*). Importantly, the siCdc5l-induced increase of Wee1 protein was negated by AAV-CDC5L, both at the protein (Fig. 4*D*) and mRNA levels (Fig. 4*E*). In contrast to simple overexpression (Fig. 4*B*), AAV-CDC5L promoted cell growth under the Cdc5l knockdown condition (Fig. 4*F*). To investigate the role of increased Wee1 by Cdc5l knockdown in cell growth, Wee1 siRNA was cotransfected with siCdc5l in ATDC5 cells. We confirmed the knockdown efficiency of Wee1 siRNA with immunoblotting (Fig. 4*G*). Similar to the effect of AAV-CDC5L, Wee1 co-knockdown partially rescued siCdc5l-mediated cell growth suppression (Fig. 4*H*). We assessed whether the expression of Cdc5l negatively associates with that of Wee1 also in mouse embryo cartilage. Indeed, double IF analysis revealed that Cdc5l and Wee1 expressions tended to be mutually exclusive (Fig. 4*I*).

### Cdc5l promotes early chondrocyte differentiation

**Figure 5:**
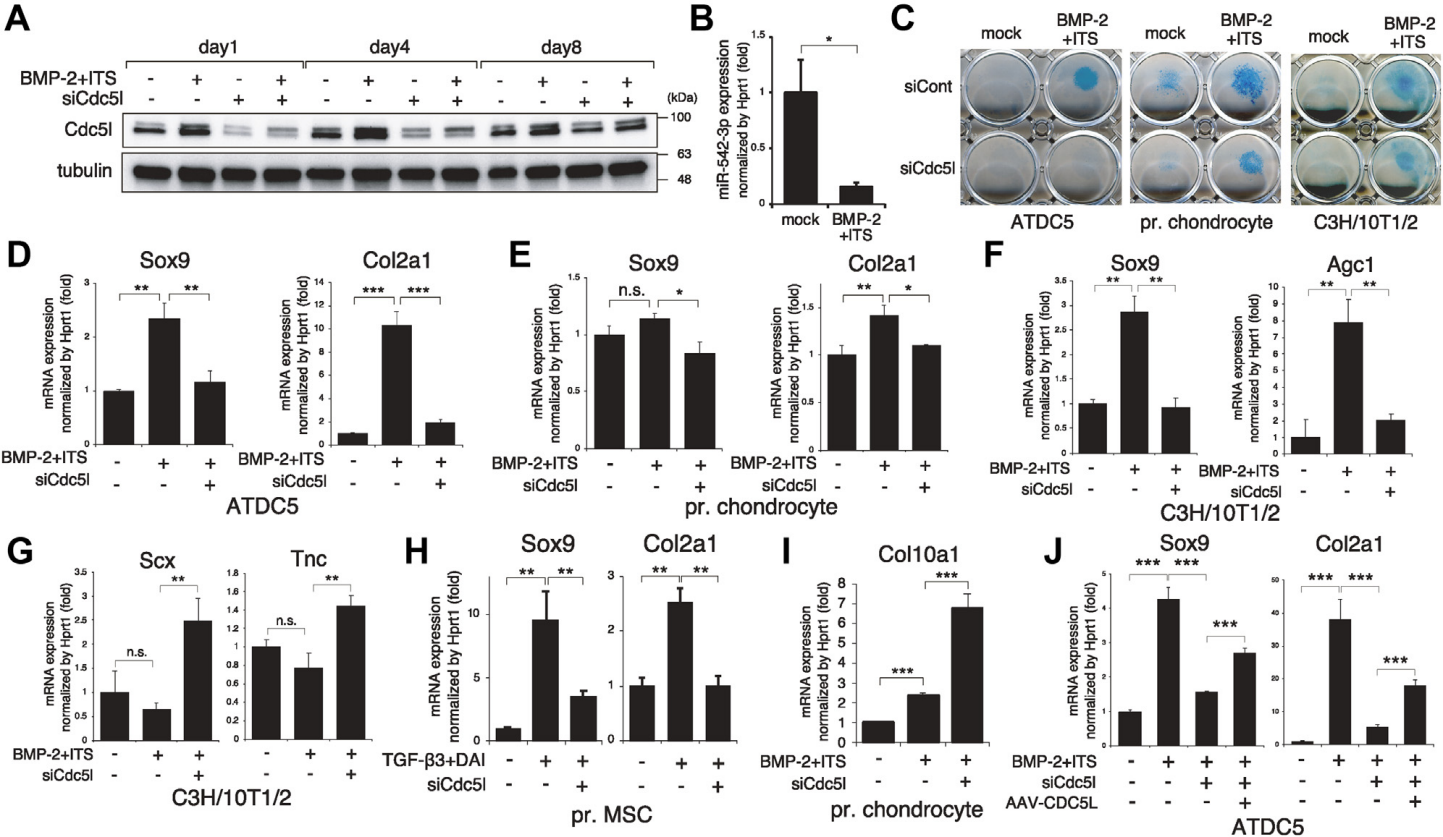
**Cdc5l promotes early chondrocyte differentiation.***A*, ATDC5 cells were transfected with siCont or siCdc5l overnight, induced with BMP-2 + ITS for the indicated time points, and subjected to immunoblotting for Cdc5l. Tubulin served as a loading control. *B*, ATDC5 cells were stimulated with or without BMP-2 + ITS for 4 days and subjected to RT-qPCR for miR-542-3p (n = 3). *C*, ATDC5 cells, primary (pr.) chondrocytes, or C3H/10T1/2 cells transfected with siCont or siCdc5l were stimulated with or without BMP-2 + ITS and subjected to alcian blue staining on day 8, 13, or 16, respectively. *D* and *E*, ATDC5 cells (*D*) or primary (pr.) chondrocytes (*E*) transfected with siCont or siCdc5l were stimulated with or without BMP-2 + ITS and subjected to RT-qPCR analysis for Sox9 and Col2a1 on day 7 or 6, respectively (n = 3). *F* and *G*, C3H/10T1/2 cells transfected with siCont or siCdc5l were stimulated with or without BMP-2 + ITS for 10 days and subjected to RT-qPCR for Sox9 and Agc1 (*F*) or Scx and Tnc (*G*) (n = 3). *H*, primary (pr.) mesenchymal stem cells (MSCs) transfected with siCont or siCdc5l were stimulated with or without TGF-β3 and a cocktail of dexamethasone, ascorbic acid, and ITS (DAI), and subjected to RT-qPCR analysis for Sox9 and Col2a1 on day 6 (n = 3). *I*, primary (pr.) chondrocytes transfected with siCont or siCdc5l were stimulated with or without BMP-2 + ITS and subjected to RT-qPCR analysis for Col10a1 on day 6 (n = 3). *J*, ATDC5 cells transfected with siCont or siCdc5l were infected with AAV-LacZ or AAV-CDC5L for 2 days, followed by BMP-2 + ITS application for 4 days. RT-qPCR for Sox9 and Col2a1 was analyzed (n = 3). ∗*p* < 0.05; ∗∗*p* < 0.01; ∗∗∗*p* < 0.001; n.s., not significant.

Next, we studied the impact of Cdc5l during early chondrocyte differentiation. We induced ATDC5 chondrocyte differentiation by applying BMP-2 with ITS supplement to the micromass culture, a system that mimics the three-dimensional structure of cartilage. Cdc5l protein was mildly increased from day 1 and further reached the peak level on day 4 (Fig. 5*A*), although it was unaffected at 2 h poststimulation (Fig. 4*D*). Because CDC5L mRNA is a known target of the microRNA miR-542-3p (28), we examined the expression of miR-542-3p and found that it was suppressed by BMP-2+ITS stimulation at day 4 (Fig. 5*B*), likely explaining the increased Cdc5l level. Alcian blue staining revealed that siCdc5l inhibited the induced cartilaginous matrix production, not only in ATDC5 cells, but also in primary chondrocytes and C3H/10T1/2 cells (Fig. 5*C*). siCdc5l also strongly eliminated the expression of the early chondrogenic markers Sox9 and Col2a1 (Fig. 5*D*). A similar but milder effect of Cdc5l knockdown was observed in mouse primary chondrocytes (Fig. 5*E*). In C3H/10T1/2 fibroblasts, siCdc5l strongly inhibited the expression of Sox9 and another early chondrogenic marker, Agc1 (Fig. 5*F*), while it increased the ligament cell marker genes scleraxis (Scx) and tenascin (Tnc) (Fig. 5*G*), indicating that C3H/10T1/2 cells lost chondrogenic features to gain ligament characters. Cdc5l siRNA exhibited a similar effect on expression of Sox9 and Col2a1 in primary MSCs, in which chondrogenesis was induced by a cocktail of transforming growth factor (TGF)-β3 plus dexamethasone/ascorbic acid/ITS (Fig. 5*H*). Because chondrocytes stop proliferating to mature into Col10a1-expressing hypertrophic chondrocytes, we checked whether siCdc5l promotes this step. Indeed, loss of Cdc5l resulted in a strong Col10a1 elevation in chondrocytes (Fig. 5*I*). To investigate the effect of forced Cdc5l expression on differentiation, we performed a rescue experiment using AAV-CDC5L in ATDC5 cells. The induction of CDC5L partially reversed the siCdc5l-induced decrease in Sox9 and Col2a1 expression (Fig. 5*J*).

### Cdc5l modulates pre-mRNA splicing of chondrogenic genes and Wee1

**Figure 6:**
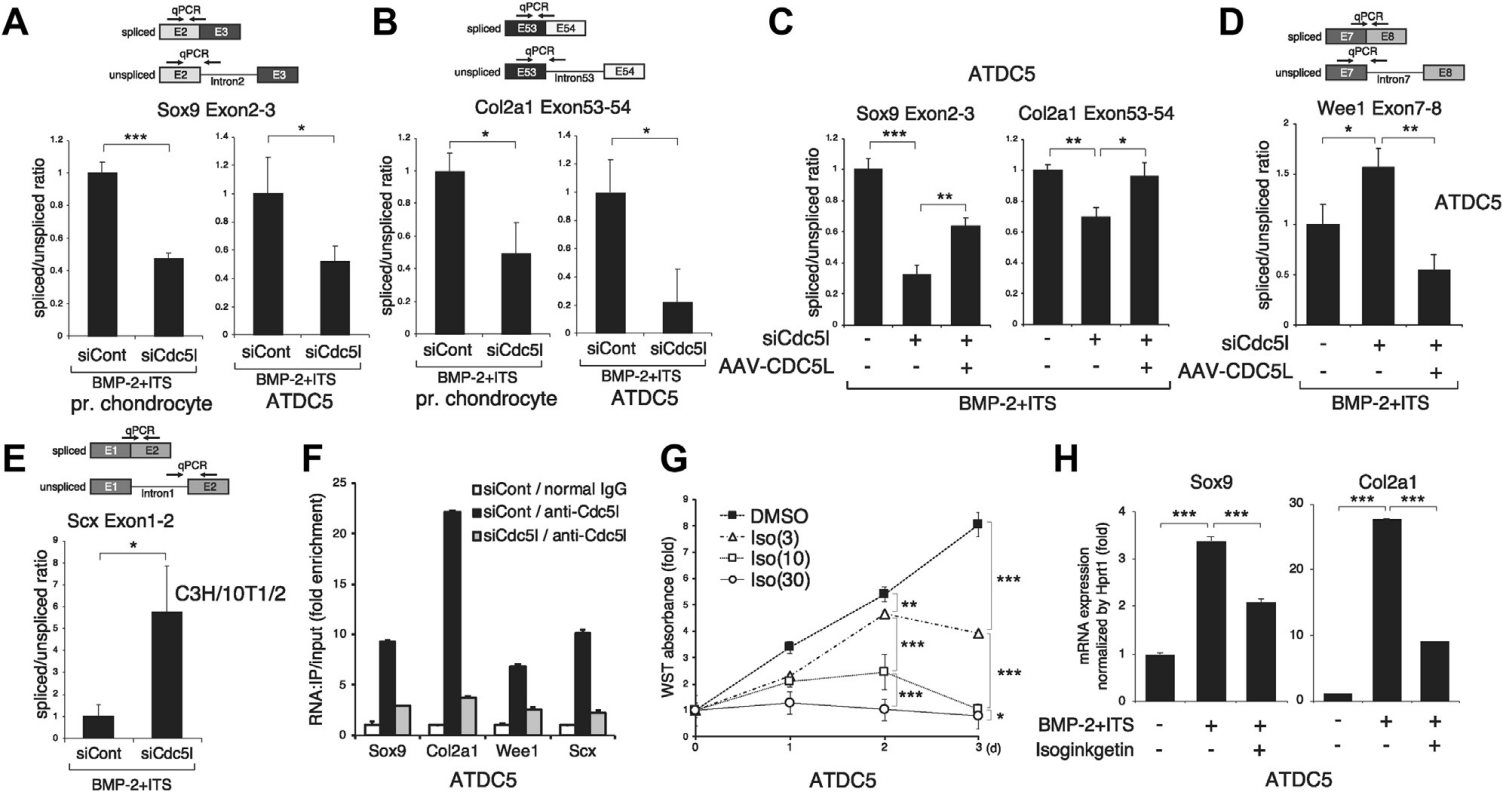
**Cdc5l modulates pre-mRNA splicing of Sox9, Col2a1, Scx, and Wee1.***A* and *B*, to evaluate the ratio of spliced *versus* unspliced mRNA of Sox9 (*A*) and Col2a1 (*B*), primary (pr.) chondrocytes or ATDC5 cells transfected with siCont or siCdc5l were treated with BMP-2 + ITS and subjected to RT-qPCR analysis (n = 3). Schematic diagrams of annealing locations of the used primers are presented (“E” denotes exon). *C*, ATDC5 cells transfected with siCont or siCdc5l were infected with AAV-LacZ or AAV-CDC5L and subjected to RT-qPCR analysis using the primer sets used in *A* and *B* to evaluate the splicing efficiency of Sox9 and Col2a1 mRNAs (n = 3). *D*, ATDC5 cells transfected with siCont or siCdc5l were infected with AAV-LacZ or AAV-CDC5L and subjected to RT-qPCR analysis to evaluate the splicing efficiency of Wee1 mRNA (n = 3); a schematic diagram of annealing locations of the primers used for detection of spliced and unspliced mRNAs is also presented. *E*, C3H/10T1/2 cells transfected with siCont or siCdc5l were treated with BMP-2 + ITS and subjected to RT-qPCR analysis to evaluate the splicing efficiency of Scx mRNA (n = 3); a schematic diagram of annealing locations of the primers is also presented. *F*, ATDC5 cells transfected with siCont or siCdc5l were stimulated with BMP-2 + ITS for 3 days and subjected to an RNA-binding protein immunoprecipitation (IP) assay with anti-Cdc5l antibody or control IgG. Immunoprecipitated and input RNA samples were subjected to RT-qPCR for the indicated genes, and the amount ratio (IP/input) was calculated as enriched RNA. qPCR was performed in triplicate. *G*, ATDC5 cells were treated with or without the pre-mRNA splicing inhibitor isoginkgetin at 3, 10, or 30 μM. Live cell numbers were monitored by water-soluble tetrazolium (WST) assay daily for 3 days (n = 8). *H*, ATDC5 cells were stimulated with or without BMP-2 + ITS in combination with or without 3 μM of isoginkgetin for 4 days, and subjected to RT-qPCR analysis for Sox9 and Col2a1 (n = 3). ∗*p* < 0.05; ∗∗*p* < 0.01; ∗∗∗*p* < 0.001.

Finally, we investigated the mechanism by which siCdc5l affected the expression of the genes tested. As CDC5L was shown to promote the pre-mRNA splicing of a set of genes involved in mitotic progression (23), we hypothesized that it is also indispensable in the pre-mRNA splicing of chondrogenic genes. We measured the relative levels of spliced and unspliced mRNAs of Sox9 and Col2a1 by RT-qPCR analyses using combinations of exon-specific, exon/exon boundary-specific, or intron-specific primers (Fig. 6, *A* and *B*). The ratio of spliced *versus* unspliced mRNAs was determined to be the pre-mRNA splicing efficiency (32). The splicing efficiencies of Sox9 and Col2a1 mRNA were substantially reduced by Cdc5l knockdown both in primary chondrocytes and ATDC5 cells (Fig. 6, *A* and *B*). If the altered gene expression is the sole result of differential splicing efficiency, the total amount of spliced and unspliced mRNA should be unchanged regardless of Cdc5l knockdown by siCdc51. Therefore, we performed conventional RT-PCR of ATDC5 or C3H/10T1/2 cell samples using primer sets targeting consecutive separate exons of Sox9, Col2a1, Wee1, and Scx and analyzed the amplicons by agarose gel electrophoresis for quantification of the band intensities (Fig. S1*A*). For Sox9 and Col2a1, although the unspliced and spliced mRNAs were increased and decreased by the presence of siCdc5l, respectively, the sum intensities of the two bands remained stable (Fig. S1*A*). The downregulated splicing efficiency of both Sox9 and Col2a1 was recovered by AAV-induced CDC5L expression (Fig. 6*C*). Because mRNA expression of Wee1 and Scx was increased by siCdc5l, we questioned whether this might be due to the enhanced pre-mRNA splicing. In contrast to Sox9 and Col2a1, siCdc5l mildly increased the Wee1 pre-mRNA splicing efficiency, and this enhancement was cancelled by AAV-CDC5L infection in ATDC5 chondrocytes (Fig. 6*D*). Moreover, the pre-mRNA splicing of Scx was also accelerated by Cdc5l knockdown, in C3H/10T1/2 cells (Fig. 6*E*). The total amounts of spliced and unspliced mRNA of the Wee1 and Scx genes were confirmed to be unchanged using conventional RT-PCR conditions (Fig. S1*A*). To clarify whether these changes in splicing efficiency were a direct consequence of Cdc5l binding to the pre-mRNA of these genes, we performed an RNA-binding protein immunoprecipitation assay using anti-Cdc5l antibody, followed by RT-qPCR on the precipitated RNA. Indeed, all of the transcripts of Sox9, Col2a1, Wee1, and Scx were shown to be bound by Cdc5l protein; of these, Col2a1 was the most enriched mRNA (Fig. 6*F*). Because unspliced transcripts are often retained in the nucleus, we determined where Cdc5l impacts RNA subcellular localization. An RNA subcellular isolation assay revealed that none of the levels of nuclear fraction mRNA of Sox9, Col2a1, Wee1, nor Scx were altered by Cdc5l knockdown (Fig. S1*B*). Finally, if mRNA splicing regulation is the major role of Cdc5l in chondrocyte proliferation and differentiation, spliceosome inhibitor compounds should show effects similar to those of Cdc5l siRNA. Indeed, treatment with isoginkgetin, a representative pre-mRNA splicing inhibitor (33), dose-dependently suppressed ATDC5 cell growth (Fig. 6*G*). Specifically, isoginkgetin inhibited the expression of Sox9 and Col2a1 in these cells (Fig. 6*H*). Thus, Cdc5l modulates pre-mRNA splicing, positively or negatively, to promote both the proliferation and differentiation of chondrocytes.

## Discussion

**Figure 7:**
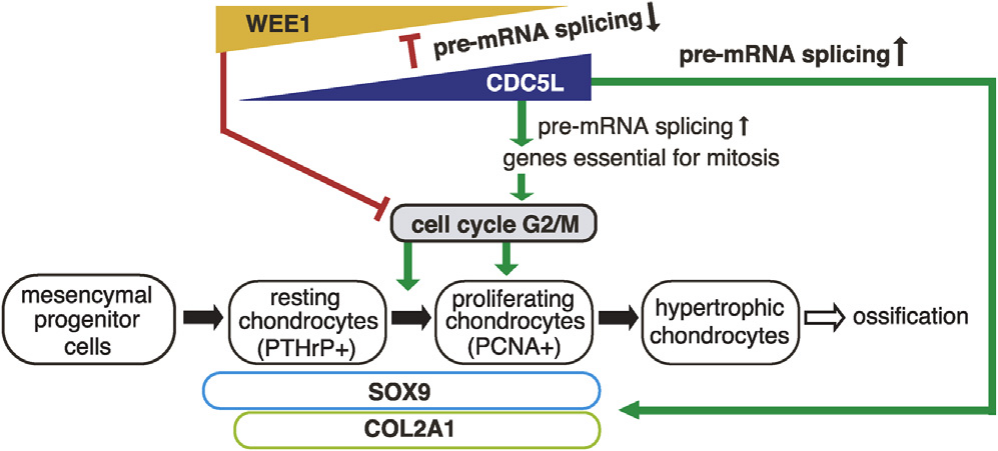
**An illustration of this study's results and a working model.** CDC5L is expressed in PCNA-positive proliferating and differentiating chondrocytes but not in hypertrophic chondrocytes. CDC5L-mediated cell cycle (G2/M) progression is essential for conversion of resting chondrocytes into proliferating chondrocytes to accelerate cell growth to enlarge the cartilage template of ossification. The inhibition of pre-mRNA splicing and subsequent expression of WEE1 by CDC5L plays a substantial part in this cell cycle acceleration. Hence, expression of CDC5L and WEE1 is mutually exclusive. Meanwhile, CDC5L promotes early chondrocyte differentiation by enhancing pre-mRNA splicing and subsequent expression of SOX9 and COL2A1. CDC5L has dual roles in early chondrogenesis, promoting cartilage growth by WEE1 suppression and enhancing early differentiation through upregulation of SOX9 and COL2A1 *via* finely tuning the splicing of the target pre-mRNAs.

Figure 7 illustrates our results and working model. CDC5L protein was expressed in PCNA-positive chondrocytic PLL cells, but not in hypertrophic chondrocyte-like PLL cells in OPLL specimens (Fig. 1). Cdc5l was also expressed in proliferating chondrocytes in mouse embryo cartilage (Fig. 2) and was accumulated in differentiating ATDC5 chondrocytes (Fig. 5*A*). Cdc5l-mediated cell cycle (G2/M) progression is essential for the conversion of resting chondrocytes into proliferating chondrocytes that enlarges the cartilage template of ossification (Fig. 3). CDC5L expression was eliminated in chondrocytes maturing into the hypertrophic stage (Fig. 1*E*), while Cdc5l knockdown promotes Col10a1 expression in mouse chondrocytes (Fig. 5*I*). In addition to its reported roles in pre-mRNA splicing of a set of genes involved in mitosis (23), Cdc5l accelerates chondrocyte proliferation by inhibiting pre-mRNA splicing and subsequent Wee1 expression (Figs. 4 and 6). Meanwhile, Cdc5l promotes early chondrocyte differentiation by upregulating pre-mRNA splicing and subsequent expression of Sox9 and Col2a1 (Figs. 5 and 6). Cdc5l directly binds to these pre-mRNAs to regulate their splicing (Fig. 6*F*). Thus, Cdc5l has dual roles in early chondrogenesis, accelerating cartilage growth by inhibiting Wee1 expression, while enhancing differentiation; both of these effects are mediated by modulating pre-mRNA splicing. This concept is supported by the results of the spliceosome inhibitor isoginkgetin mimicking the inhibitory effect of siCdc5l on cell proliferation as well as the expression of Sox9 and Col2a1 in ATDC5 chondrocytes (Fig. 6, *G* and *H*).

Although the endochondral ossification process had been suggested to take place during OPLL pathogenesis, the mechanism by which the degenerative PLL cells undergo proliferation (hyperplasia) before ossification had remained unclear. In this study, we showed CDC5L and PCNA expression in chondrocytic fibroblasts in OPLL, while the OPLL risk-allele C of the susceptibility SNP (rs927485) was associated with higher CDC5L expression levels in human fibroblasts (Fig. 1). The SNP rs927485 does not reside in the CDC5L gene itself, but lies in the vicinity of CDC5L; the two display linkage disequilibrium (16). This SNP likely acts as an enhancer for CDC5L gene transcription. Furthermore, we observed a positive role of Cdc5l in mouse chondrocyte growth *in vitro* (Fig. 3). Although CDC5L is involved in pre-mRNA splicing to promote the expression of mitosis-related genes (23), the precise target genes essential for the complex G2/M transition process are not fully elucidated. Cell cycle regulation is fine-tuned by the cooperation of cyclins, cyclin-dependent kinases (CDKs), and CDK inhibitors. Cyclin/CDK complexes activate cell cycle proteins essential for transition to the next cell cycle phase, while CDK inhibitors interfere with cyclin/CDK complex formation (34). The decision to enter mitosis primarily depends on CDK1 activity after the initiation of the G2/M transition, and CDK1 and cyclin B form a complex to become part of the M-phase promoting factor (35, 36). Importantly, CDK1 activity is negatively regulated by WEE1, which catalyzes inhibitory phosphorylation of tyrosine 15 on CDK1 (37). Indeed, Cdk1 is indispensable for the maintenance of chondrocyte proliferation in mouse bone growth plate (38). These findings are in line with our results, which showed that expression of Cdc5l, not Wee1, was detected in proliferating chondrocytes along with Pcna expression in the mouse embryo bone, while Cdc5l and Wee1 showed mutually exclusive distribution (Fig. 4*I*). As Cdc5l siRNA-induced cell growth suppression was partially rescued by Wee1 knockdown (Fig. 4*H*), Wee1 is suggested to be a major target of Cdc5l in the G2/M transition. While Cdc5l knockdown suppressed the cell cycle, CDC5L overexpression in ATDC5 chondrocytes also inhibited cell growth by inducing apoptosis (Fig. 4). This forced CDC5L expression strongly eliminated Wee1 expression (Fig. 4). In response to DNA damage, WEE1 negatively modulates cell entry into mitosis *via* regulation of the G2/M checkpoint through CDK1 phosphorylation, which provides cells with a survival advantage that allows time to repair the damaged DNA (30, 37, 39). WEE1 inhibition abrogates the G2/M checkpoint, forcing cells carrying DNA damage to enter unscheduled mitosis to undergo apoptosis, referred to as mitotic catastrophe (40). For example, MK1775, a small selective kinase inhibitor compound of WEE1, induces unscheduled mitotic entry and apoptosis in sarcoma cells (41). Therefore, AAV-CDC5L-induced apoptosis is suggested to be a result, at least in part, of the excessively eliminated expression of Wee1. However, AAV-CDC5L increased the cell number under the Cdc5l-silenced condition (Fig. 4*F*), in which the AAV-CDC5L-infected cells maintained steady-state Wee1 expression levels (Fig. 4, *D* and *E*). Hence, the expression levels of CDC5L and WEE1 should be finely maintained to avoid apoptosis during early chondrogenesis.

Regarding chondrocyte differentiation, R-spondin 2 (RSPO2), another OPLL susceptibility gene identified by GWAS, was demonstrated to inhibit chondrocyte differentiation of ATDC5 cells by promoting canonical Wnt signaling, while the risk allele of the corresponding susceptible SNP rs374810 was associated with RSPO2 downregulation in human fibroblasts (42). Therefore, the common output phenotype obtained from RSPO2 inhibition and CDC5L activation is the promotion of early chondrogenesis, which strongly supports the notion that OPLL is formed through endochondral ossification. However, the fact that Cdc5l enhanced both proliferation and differentiation of chondrocytes is contradictory, because generally cells exit the cell cycle when they undergo differentiation. For example, loss of Cdk1 reduces the proliferation and accelerates the differentiation of chondrocytes (38), and Cdk1 activation *via* suppression of inhibitory tyrosine 15 residue phosphorylation decreases Sox9 and Col2a1 expression in ATDC5 cells (43). Interestingly, but not surprisingly considering the function of CDC5L as a spliceosome complex component, Cdc5l was indispensable for the pre-mRNA splicing of early chondrogenic genes in mouse chondrocytes. Thus, Cdc5l is the unique nuclear protein that drives both early chondrocyte differentiation and proliferation. As its physiological roles in the endochondral ossification process *in vivo* remain to be elucidated, cartilage-specific Cdc5l knockout mice should be investigated in the future.

In contrast to chondrogenic genes, loss of Cdc5l in C3H/10T1/2 fibrocytes enhanced expression of the ligament cell markers Scx and Tnc and promoted mRNA splicing (Figs. 5, *G* and 6, *E*). Moreover, Wee1 mRNA splicing and expression were also increased by Cdc5l knockdown (Figs. 4, *D* and *E*, 6D). These results indicate that CDC5L not only promotes pre-mRNA splicing but can also interfere with it in a context-dependent manner. It was reported that after transfection of CDC5L siRNA in HeLa cells, 407 genes were decreased and 447 genes were increased (23), suggesting that CDC5L plays both positive and negative roles in pre-mRNA splicing. Therefore, specific partners of CDC5L might exist in the spliceosome complex that fine-tune the mRNA splicing of certain genes, positively or negatively. However, in addition to being an mRNA splicing regulator, CDC5L can function as a DNA-binding transcription factor to regulate target gene expression (28, 29, 44). Therefore, it should be noted that we were careful not to rule out the possible actions of CDC5L as a transcription factor in chondrogenesis.

The increased expression of CDC5L in degenerated PLL cells in OPLL is suggested to eliminate the ligament phenotype and assign the chondrogenic phenotype for the initiation of endochondral ossification. The possibility that miR-542-3p (Fig. 5*B*) might be downregulated in degenerated PLL cells in OPLL should be investigated in the future. Regarding OPLL prevention or therapy, inhibiting CDC5L activity may be useful in eliminating the chondrogenesis of PLL. However, because CDC5L modulates pre-mRNA splicing of a wide range of genes, it is itself an inappropriate target. Rather, the chondrocyte-specific and gene-specific CDC5L cofactors in the spliceosome complex should be identified as therapeutic targets. In conclusion, we demonstrated CDC5L/Cdc5l expression in proliferating chondrocytes in OPLL or mouse embryo bone growth plates and Cdc5l-mediated promotion or inhibition of the pre-mRNA splicing of early chondrogenic genes (Sox9 and Col2a1) or Wee1, respectively. These dual roles of CDC5L in cell proliferation and differentiation might cooperatively promote the initiation phase of endochondral ossification.

## Experimental procedures

### Human and mouse subjects

All the fibroblast donor individuals gave written informed consent to participate in the expression quantitative trait loci analysis study. This analysis was approved by the Ethical Committees at the RIKEN Yokohama Institute (17-16-39(2)). We obtained human OPLL samples from three patients who underwent anterior decompressive surgery. Written informed consent was collected from all the patients. This study was approved by the Ethics Committee of Kagoshima University (number 28-21). These studies abide by the Declaration of Helsinki principles. Mouse experiments were approved by the Institutional Animal Care and Use Committee of Kagoshima University (MD18015).

### Expression quantitative trait loci analysis

Human fibroblast cells were cultured in Dulbecco's modified Eagle's medium (DMEM) containing 10% fetal bovine serum (FBS). Total RNA was extracted with spin-vacuum (SV) total RNA isolation system (Promega), according to the manufacturer's instructions. cDNA was synthesized from total RNA using the Sensiscript RT kit (Qiagen). Quantitative real-time PCR was performed with a StepOnePlus real-time PCR system (Applied Biosystems) using QuantiTect SYBR Green PCR kit (Qiagen). The value of CDC5L mRNA expression was normalized to β-actin in the same sample. Genomic DNA was extracted from fibroblasts using the NucleoSpin tissue kit (Takara Bio, Kusatsu). SNP rs927485 was genotyped by a PCR-based invader assay (Third Wave Technologies) (45). The correlation between gene expression values transformed to the log_2_ scale and the number of an allele of rs927485 was evaluated with Pearson's correlation coefficient and tested with a linear regression model.

### Immunohistochemistry (IHC)

Forelimbs removed from mouse embryos at 17.5 days postcoitum (E17.5) were fixed overnight in 4% paraformaldehyde in phosphate-buffered saline (PBS) to be embedded in paraffin blocks. The sections of human OPLL samples or mouse embryo bones were deparaffinized and subjected to antigen retrieval by incubation with L.A.B. solution (Polysciences). Endogenous peroxidase was inactivated by 3% H_2_O_2_ in PBS. The cell membrane was permeabilized with 0.2% Triton-X-100 in PBS. Nonspecific antigens on the samples were blocked by 10% normal goat serum for 60 min. The primary antibodies used were rabbit polyclonal anti-CDC5L (1:100, IHC-00605, BETHYL Laboratories), rabbit polyclonal anti-CDC5L (1:200, HPA011361, ATLAS Antibodies), mouse monoclonal anti-PCNA (1:10,000, ab29, Abcam), rabbit polyclonal anti-SOX9 (sc-20095, H-90, Santa Cruz Biotechnology), rabbit polyclonal anti-COL II (1:200, LS-C175971, LifeSpan Biosciences), and rabbit polyclonal anti-COL X (1:200, 14-9771-82, clone X53, Thermo Fisher Scientific). Normal rabbit IgG (sc-2027, Santa Cruz Biotechnology) or normal mouse IgG (sc-2025, Santa Cruz Biotechnology) served as negative controls for primary antibodies. Histofine Simple Stain MAX-PO (MULTI) (424151, Nichirei Biosciences), a mixture of anti-mouse and anti-rabbit peroxidase-conjugated secondary antibodies, was applied to detect the signals. Signals were visualized using DAB solution (415171, Nichirei Biosciences). Counterstaining was performed with Mayer's hematoxylin solution. Images were captured using the BZ-X710/BZ-X700 microscope system (Keyence).

### Immunofluorescence (IF)

Sections of human OPLL samples or mouse embryo bones were deparaffinized and incubated with L.A.B. solution for antigen retrieval. After incubations with 3% H_2_O_2_ and 0.2% Triton-X-100, specimens were blocked with CAS block (Invitrogen). For double IF for CDC5L and PCNA, specimens were incubated overnight with anti-CDC5L rabbit polyclonal antibody (1:200, BETHYL Laboratories, or 1:50, ATLAS Antibodies), followed by incubation with anti-rabbit IgG Alexa Fluor 488 (1:500, A11008, Invitrogen) for 60 min. Subsequently, samples were blocked with CAS solution, then incubated with anti-PCNA mouse antibody (1:200 or 1:5000, Abcam) for 60 min, followed by incubation with anti-mouse IgG Alexa Fluor 568 (1:500, A11061, Invitrogen) for 60 min. For double IF of CDC5L and WEE1, anti-WEE1 rabbit antibody (1:200, #13084, Cell Signaling Technology, Danvers, MA), anti-rabbit IgG Alexa Fluor 568 (1:500, A11011, Invitrogen), anti-CDC5L mouse antibody (1:50, LS-C342413, LifeSpan Biosciences), and anti-mouse IgG Alexa Fluor 488 (1:500, A11001, Invitrogen) were used. Cell nuclei were stained with Hoechst 33342 (1:1000, H3570, Invitrogen). Images of immunofluorescence were captured with the BZ-X710/BZ-X700 microscope system.

### Cell cultures and cell lines

The chondrogenic cell line ATDC5 was obtained from RIKEN BioResource Center (Tsukuba). The cells were maintained in DMEM/Ham's F-12 (1:1) (Invitrogen) containing 5% FBS, 100 units/ml penicillin G, and 100 μg/ml streptomycin. The mouse C3H/10T1/2 cell line was obtained from the American Type Culture Collection (ATCC), which were maintained in Eagle's basal medium (Sigma-Aldrich) with 2 mM L-glutamine, 10% FBS, and antibiotics. Primary C57BL/6J mouse chondrocytes were isolated from new-born mice. Briefly, articular cartilage of the femoral heads, femoral condyles, and tibia plateaus was isolated and digested by 3 mg/ml collagenase D (Roche) for 45 min, followed by 0.5 mg/ml collagenase D overnight. The chondrocytes were filtered through a sterile 40 μm cell strainer and cultured in DMEM/Ham's F-12 (1:1) containing 10% FBS and antibiotics. C57BL/6 mouse MSCs were obtained from Cyagen Biosciences and cultured in OriCell mouse MSC complete growth medium (Cyagen Biosciences). For expression analysis of differentiation markers, micromass culture was performed as previously described (46). For chondrogenic differentiation, BMP-2 (Peprotech) was applied at a concentration of 100 ng/ml with ITS supplement (Sigma-Aldrich) for ATDC5, C3H/10T1/2, or primary chondrocytes, while transforming growth factor (TGF)-β3 (R&D Systems, Minneapolis, MN) was applied at 5 ng/ml with ITS, 100 μmol/L ascorbic acid 2-phosphate (Sigma-Aldrich), and 100 nmol/L dexamethasone (Sigma-Aldrich) for MSCs.

### RNA interference

Dharmacon siRNA ON-TARGETplus SMARTpool, a mixture of four independent siRNAs of mouse Cdc5l (L-054807-01), mouse Wee1 (L-040623-00), and the nontargeting control siRNA (D-001810-10) were purchased from Thermo Fisher Scientific. The siRNAs were transfected into cells using Lipofectamine RNAiMax (Invitrogen).

### Adeno-associated virus (AAV)

The AAVpro 293T cell line (632273, Takara Bio) was transfected with the FLAG-tagged human CDC5L vector, pHelper vector, and pRC6 vector (AAV pro Helper free system, Takara Bio) using TransIT-293 Transfection Reagent (MIR2705, Mirus Bio). After 48 h, cells were detached by the addition of 0.5 M ethylenediaminetetraacetic acid (EDTA, pH 8.0) and then pelleted by centrifugation. The cell pellet was lysed with AAV extraction solution A and centrifuged, and then AAV extraction solution B was added to the supernatant. The collected AAV was purified with the AAV pro purification Kit (Takara Bio), and the viral titer was calculated using the AAV titration kit (Takara Bio). ATDC5 cells were infected with AAV-LacZ or AAV-ZsGreen at a multiplicity of infection of 1 × 10^6^ viruses/cell.

### Real-time and conventional RT-PCR

Cultured cells were lysed with the TRIzol reagent (Invitrogen) to purify RNA, and 1 μg of total RNA was reverse transcribed into cDNA using the Verso cDNA Kit (Thermo Fisher Scientific). For microRNAs, the Mir-X miRNA first-strand synthesis kit (Takara Bio) was employed. The relative amounts of gene transcripts were determined by real-time qPCR analysis using TB green premix Ex Taq II (Takara Bio) and the Thermal Cycler Dice TP850 system (Takara Bio). PCR was performed in duplicate per sample, and the measured expression level of each gene was normalized to that of Hprt1. For conventional RT-PCR, PCR was performed using Ex Taq hot start version (Takara Bio) on the GeneAmp PCR System 9700 (Applied Biosystems), and the amplicons were separated by agarose electrophoresis for analysis by the Image J software (https://imagej.nih.gov/ij/). The primers used in PCR reactions are listed in Table S1.

### Immunoblotting

Cells were lysed in M-PER lysis buffer (Thermo Fisher Scientific) supplemented with aprotinin, sodium orthovanadate, and phenylmethylsulfonyl fluoride. After centrifugation, the supernatants were mixed with sodium dodecyl sulfate (SDS) loading buffer. SDS–polyacrylamide gel electrophoresis, membrane transfer, blocking, and chemiluminescence were performed according to the standard protocol. Blots were incubated with the following primary antibodies: anti-CDC5L (1:500, HPA011361, ATLAS), anti-WEE1 (1:1000, #13084, Cell Signaling Technology), anti-FLAG (1:1000, F7425, Sigma-Aldrich), or anti-tubulin (1:1000, DM1A, T9026, Sigma Aldrich). Then, the signal was detected with horseradish peroxidase-conjugated secondary anti-rabbit or anti-mouse antibodies (1:10,000, Cell Signaling Technology). Chemiluminescence signals were captured using the LAS 4000 Mini Image Analyzer (Fujifilm). Each immunoblot experiment was performed three times, and the representative images are presented.

### Cell growth analysis

Cells (1000/well) were seeded onto 96-well plates (n = 8). At the indicated time points, the cells were subjected to water-soluble tetrazolium (WST)-1 cell proliferation assay (Roche) according to the manufacturer's protocol. The absorbance was measured at 450 nm on a microplate reader.

### Alcian blue staining

Cultured cells were rinsed with PBS and fixed in 4% paraformaldehyde for 10 min at room temperature. After incubation in 0.1 N HCl for 10 min, cells were stained with 0.5% Alcian blue 8GX (Sigma-Aldrich, A5268) in 0.1 N HCl overnight.

### Cell cycle analysis

For thymidine double block, cells were seeded at 2 × 10^5^ cells/well in 6-well plates and treated with thymidine (Nacalai tesque) at 2 mM for 14 h. After washing in normal medium for 12 h, cells were treated with 2 mM thymidine for another 14 h. The cells were harvested at 2 h intervals up to 12 h and 26 h after release. The harvested cells were fixed with 75% ethanol at −20 °C for at least 2 h and collected by centrifugation and resuspended with Guava cell cycle reagent (Merck Millipore). DNA content was analyzed using a flow cytometry analyzing system CytoFLEX (Beckmann Coulter).

### Embryonic cartilage organ culture and lentiviral (LV) infection

Metatarsal bone rudiments were harvested from C57BL/6J mouse embryos at E15.5 or E17.5 and cultured in DMEM with 10% FBS and antibiotics. Images of the cultured cartilages were captured daily to measure the length using the BZ-X710/BZ-X700 microscope system (Keyence). The CopGFP control lentiviral particle (LV sh-Control, sc-108084) and Cdc5l shRNA lentiviral particle (LV sh-Cdc5l, sc-62088-V) were purchased from Santa Cruz Biotechnology. ATDC5 cells were infected with LVs following the manufacturer's protocol. Briefly, cells were cultured in 6-well plates in complete medium. After 24 h, the medium was removed and substituted with 2 ml of medium containing polybrene (sc-134220, Santa Cruz) at a final concentration of 5 μg/ml. Then, cells were infected by adding the sh-Control or sh-Cdc5l lentiviral particles to the culture, mixed by swirling, and incubated overnight. The next day, the medium was changed with fresh complete medium, and cells were cultured for 2 days for harvest. In mouse embryonic bone organ cultures, E15.5 metatarsals were infected by adding 20 μl of LV sh-Control or LV sh-Cdc5l lentiviral particles with 5 μg/ml polybrene in 1.5 ml tubes and incubated overnight. The next day, the lentiviral particles were removed and cultured in complete medium with or without BMP-2.

### TUNEL assay

Apoptosis was detected by analyzing DNA fragmentation using the terminal deoxynucleotidyl transferase dUTP nick-end labeling (TUNEL) assay with the *in situ* apoptosis detection kit (Takara Bio).

### RNA-binding protein immunoprecipitation and RNA subcellular fraction isolation

Two 100-mm dishes of ATDC5 cell culture per sample, transfected with siCont or siCdc5l, were stimulated with BMP-2 and ITS for 3 days and then subjected to RNA immunoprecipitation (IP) using a Magna RIP kit (Merck Millipore) with anti-CDC5L rabbit polyclonal antibody (BETHYL Laboratories) or control IgG, according to the manufacturer's protocol. Immunoprecipitated and input RNA samples were subjected to RT-qPCR, and the amount ratio (IP/input) was calculated as enriched RNA. For RNA subcellular isolation, ATDC5 cells transfected with siCont or siCdc5l were stimulated with BMP-2 and ITS for 3 days followed by fractionation using kit #25501 (Active Motif), according to the manufacturer's manual. Purified nuclear, cytoplasmic, and total RNA were subjected to RT-qPCR.

### Inhibitor compound

The pre-mRNA splicing inhibitor isoginkgetin (HY-N2117, MedChemExpress) was dissolved in dimethyl sulfoxide and applied at 3, 10, and 30 μM.

### Statistical analysis

The results are expressed as the mean ± standard deviation of at least three independent experiments. Statistical comparisons between various treatments were performed using the unpaired Student's *t* test. *p* < 0.05 was considered statistically significant.

## Data availability

All data required for the conclusions made here are present within the manuscript.

## Supporting information

This article contains supporting information.

## Conflict of interest

The authors declare that they have no conflicts of interest with the contents of this article.
