# Supplementary material for: CDC5L promotes early chondrocyte differentiation and proliferation by modulating pre-mRNA splicing of SOX9, COL2A1, and WEE1
Source: J Biol Chem. 2021 Jul 21;297(2):100994. doi: 10.1016/j.jbc.2021.100994 (PMC8363834; doi:10.1016/j.jbc.2021.100994)
Supplement: Figure S1 and Table S1 [file mmc1.pdf]

Supporting information

CDC5L promotes early chondrocyte differentiation and proliferation by modulating pre-mRNA splicing of *SOX9*, *COL2A1*, and *WEE1*

Go Jokoji,[1,2] Shingo Maeda,[1*] Kazuki Oishi,[2] Toshiro Ijuin,[1,2] Masahiro Nakajima,[3] Hiroki Tawaratsumida,[1,2] Ichiro Kawamura,[2] Hiroyuki Tominaga,[2] Eiji Taketomi,[4] Shiro Ikegawa,[3] and Noboru Taniguchi[1,2]

[1]Department of Bone and Joint Medicine, Graduate School of Medical and Dental Sciences, Kagoshima University, Kagoshima, Japan
[2]Department of Orthopaedic Surgery, Graduate School of Medical and Dental Sciences, Kagoshima University, Kagoshima, Japan
[3]Laboratory for Bone and Joint Diseases, RIKEN Center for Integrative Medical Sciences, Tokyo, Japan
[4]Department of Orthopaedic Surgery, Japanese Red Cross Kagoshima Hospital, Kagoshima Japan

<u>List of supporting material</u>

1. Supplementary Table S1
2. Supplementary Figure S1

Supplementary Table S1

RT-qPCR primers for evaluating gene expression		
Gene	Forward (5' to 3')	Reverse (5' to 3')
Cdc5l	TCTGCCACAGGAGATCGAGTTG	ACCACGGTGCTGGGTAACTTGT
Sox9	TCTGGAGGCTGCTGAACGAGA	TCTTCGGCCTCCGCTTGTC
Agc1	GGTGGCGCTGTAACAAACTTCC	ACACCAGCCTCAGGGTAAGCAG
Col2a1	ACTGTCCCTCGGAAAAACTGG	TCGCCATAGCTGAAGTGGAAG
Col10a1	TTCAGGGAGTGCAATCATGGAG	GCAATTGGAGCCATACCTGGTC
Pthrp	GAGTGCTGCCGCCAAGACTAAT	CGAAAACCGGGCGTTTTACATA
Scx	CGGCGATTCGAAGTTAGAAGGA	TCCGTGACTCTTCAGTGGCATC
Tnc	ATGCTGATGGCCTAGAGATGCAG	GTTTTCCAAGATGGCGAACACAC
Wee1	TTCACAGATCGGATGGCCACTA	GGCTGACAGAGCGGTTCATTTT
Hprt1	CGTTGGGCTTACCTCACTGCTT	CAAAAAGCGGTCTGAGGAGGAA

RT-qPCR primers for detecting spliced/unspliced mRNA			
Gene	Target exon or intron	Direction	5' to 3'
Sox9	exon 2	forward	GAAGTCGGTGAAGAACGGACAA
Sox9	exon 2/3 boundary	reverse	CTGAGATTGCCCAGAGTGCTC
Sox9	intron 2	reverse	GCACCTTAAGAGTGTGCCAGGAG
Col2a1	exon 53	forward	CTCATCCAGGGCTCCAATGATGTA
Col2a1	exon 53/54 boundary	reverse	TACCAGTGTGTTTCGTGCAG
Col2a1	intron 53	reverse	GACTCAAGCCTCTAATGAGTCCCT
Scx	exon 1/2 boundary	forward	CCTCAGCAACCAGAGAAAGTTGAG
Scx	exon 2	reverse	TGTGGACCCTCCTCCTTCTAACTT
Scx	intron 1	forward	AGTGCTGGGTGACACTAAGTCT
Wee1	exon 7	forward	TCCCAAATGCTGTCTCTGAGGA
Wee1	exon 7/8 boundary	reverse	TTGTTACATGCCCAAGATCACCT
Wee1	intron 7	reverse	TCTTTCCCACCACACCCACA

RT-PCR primers for detecting spliced/unspliced mRNA			
Gene	Target exon or intron	Direction	5' to 3'
Sox9	exon 2	forward	AGAGGCCACGGAACAGACTCAC
Sox9	exon 3	reverse	ATGACGTCGCTGCTCAGTTCAC
Col2a1	exon 53	forward	CAACACCGCTAACGTCCAGATG
Col2a1	exon 54	reverse	AGCAGACAGGCCCTATGTCCAC
Scx	exon 1	forward	CTCCAGTCCGAACACATGTGCCCG
Scx	exon 2	reverse	TGTTCGGCTGCTTAGAGTCAAGCC
Wee1	exon 4	forward	CTGAAAGCAACATGAAGTCACGGT
Wee1	exon 5	reverse	CACAGCGTGAGCATACACTTCTCT
Hprt1	exon 8	forward	GCCCTTGACTATAATGAGTACTTC
Hprt1	exon 9	reverse	TGACAACGATTTACTGAAAGTGGG

RT-qPCR primers for detecting microRNA		
miR	Forward (5' to 3')	Reverse
miR-542-3p	TGTGACAGATTGATAACTGAAA	mRQ 3' primer supplied in the kit

Supplementary Figure S1

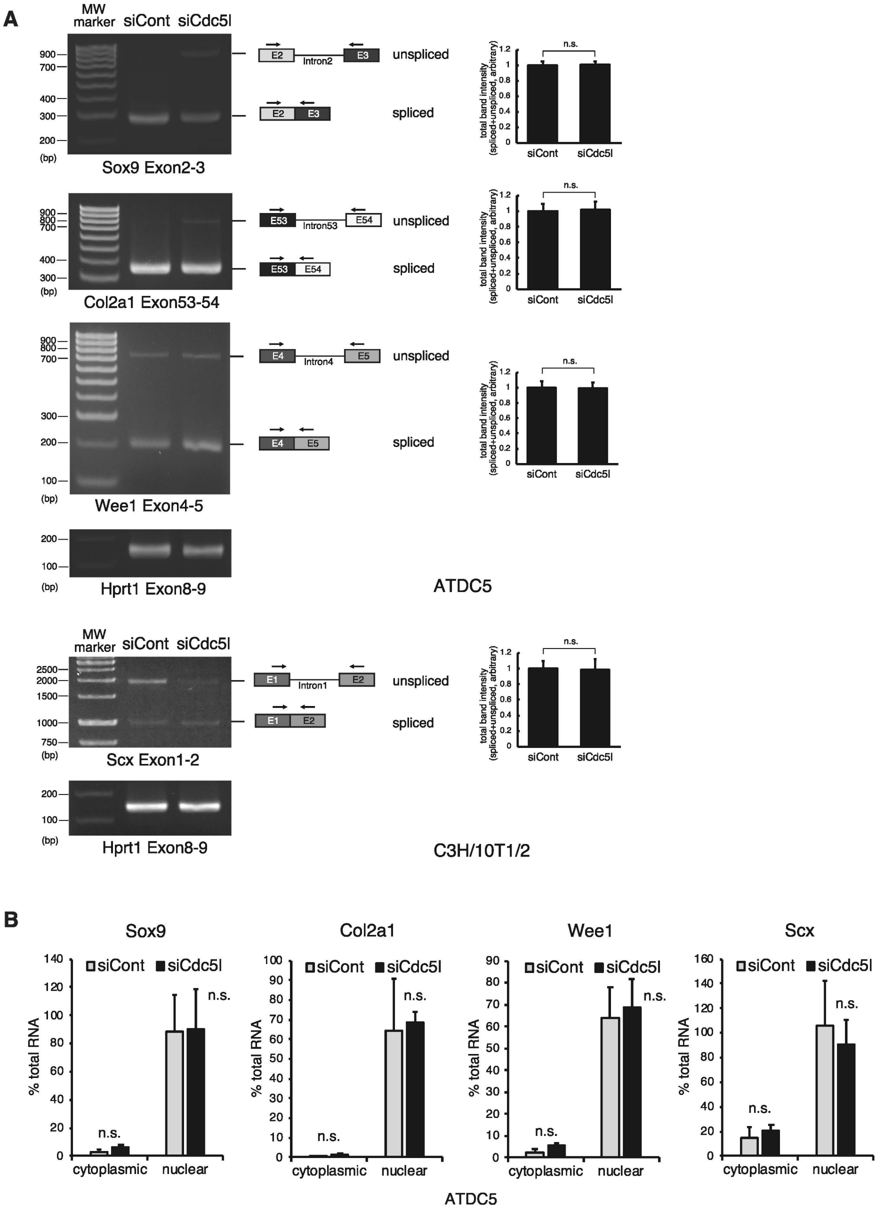

Supplementary Figure S1.

A, ATDC5 cells or C3H/10T1/2 cells transfected with siCont or siCdc5l were treated with BMP-2 + ITS and subjected to RT-PCR analysis. Schematic diagrams of annealing locations of the used primers are presented ("E" denotes exon). The intensity of total (unspliced plus spliced) mRNA PCR products was measured by the Image J software (n=3, right panels). *B*, ATDC5 cells transfected with siCont or siCdc5l were treated with BMP-2 + ITS for 3 days to be subjected to RNA subcellular fraction isolation, and the purified nuclear, cytoplasmic and total mRNA was subjected to RT-qPCR analysis. Each subcellular fraction was plotted as a percentage of total RNA.
